# Editorial: Impact of uncontrolled diabetes on oral disease progression and healing

**DOI:** 10.3389/fdmed.2024.1487051

**Published:** 2024-09-11

**Authors:** Ryan Moseley, Rachel Jane Waddington

**Affiliations:** ^1^ Disease Mechanisms Group, School of Dentistry, College of Biomedical and Life Sciences, Cardiff University, Cardiff, United Kingdom; ^2^ Biomaterials Group, School of Dentistry, College of Biomedical and Life Sciences, Cardiff University, Cardiff, United Kingdom

**Keywords:** type 2 diabetes (DM2), oral disease and systemic disease, bone healing, inflammation, oxidative stress, antioxidants

**Editorial on the Research Topic**
Impact of uncontrolled diabetes on oral disease progression and healing

It is increasingly becoming established that uncontrolled type I and type II diabetes can have significant deleterious effects on normal metabolism and cellular functions in a range of tissues, including those within the oral cavity, leading to the initiation, progression and persistence of various oral-related clinical conditions (1–4).

The aim of the Research Topic was to disseminate new preclinical-, clinical- and public health-related evidence which highlighted the relationship between diabetes and its impact on certain oral manifestations associated with hard and soft tissues, including periodontal disease, caries and osseointegration, whilst further providing scope and perspectives into the development of novel therapeutic strategies aimed at alleviating or preventing these oral conditions in diabetic patients in future.

Within this Research Topic, three research articles and three review articles were accepted, which addressed various topics relevant to oral disease aetiology and pathophysiology, including the impact of diabetes on the oral microbiome and host immune responses (de Cássia Negrini et al.), and impaired bone repair relevant to periodontal healing and osseointegration as a consequence of altered transforming growth factor-β_1_ (TGF-β_1_) signalling and extracellular matrix deposition (Yusop et al.) or due to imbalances in oxidative stress and cellular antioxidant levels (Li et al.). Additional clinical studies further compared the prevalence of dental caries and associated factors among diabetic and nondiabetic patients (Shiferaw et al.) or aimed to identify the most appropriate treatment for periodontitis (Dallaserra et al.) and other oral conditions (de Cássia Negrini et al.), ultimately addressing the negative impact that diabetes can have on oral health status and related quality of life in diabetic patients (Homagarani et al.).

From the mechanistic studies published, the consensus was that uncontrolled diabetes and associated hyperglycaemia stimulated significant changes in many facets of the pathological disease and healing processes in affected individuals, inducing alterations in the oral microbiome, leading to increased periodontal pathogenicity and dysfunctional immune-inflammatory responses (de Cássia Negrini et al.). Similarly, bone healing was severely delayed in diabetic animal models *in vivo*, identified to be associated with significantly elevated TGF-β_1_ levels within the bone extracellular matrix (Yusop et al.). Laboratory studies further showed that such events could be a possible consequence of elevated TGF-β_1_ secretion by mesenchymal stromal cells (MSCs) and osteoblasts, coupled with elevated decorin deposition, which disrupts TGF-β_1_ bioavailability, reparative M2 macrophage formation and cell signalling during normal bone repair. A review article also described the current evidence to support a contributary role for excessive reactive oxygen species (ROS) generation and oxidative stress in the pathogenesis of impaired bone healing in diabetic patients, at the expense of depleted cellular/tissue antioxidant defences (Li et al.). The article further proposed the underlying cell signalling mechanism disrupted in MSCs, osteoblasts and endothelial cell, which lead to impaired angiogenesis and overall bone repair in these patients. Therefore, these studies collectively concluded that poor glycaemic control exerts detrimental effects on oral tissues, leading to the onset and exacerbation of various disease manifestations.

From a clinical and public health perspective, a study by (Shiferaw et al.) identified that the prevalence of dental caries significantly increased in diabetic patient cohorts, vs. their matched non-diabetics counterparts, associated with risk factors such as poor oral hygiene, dry mouth and elevated sugar intake. Furthermore, the study by (Dallaserra et al.) determined that there is lack of scientific information about risk of infectious or systemic complications in decompensated diabetic patients as a consequence of periodontal treatment, and whether antibiotic treatment or prophylaxis reduced the risk of infection complications occurring in these patients.

From future interventional viewpoint, the published studies strongly highlight that oral healthcare provision is paramount (de Cássia Negrini et al.), coupled with the need for additional oral healthcare instruction/education in preventive measures, such as dental cleaning practices and diet (Shiferaw et al.), in diabetic patients to improve oral hygiene. However, despite diabetes causing functional limitations, physical pain, and psychological discomfort, it has been proposed that diabetes no statistical significant impact on the quality of life in diabetic patients (Homagarani et al.).

The published studies also supported novel approaches to alleviate the severity of the effects of uncontrolled hyperglycaemia on bone healing, through the application of exogenous antioxidant-based therapeutic entities which have shown to be effective in alleviating oxidative stress and inflammation, whilst promoting bone repair, during numerous preclinical studies (Li et al.). Thus, such novel antioxidant strategies may ultimately aid the prevention and/or treatment of such oral conditions in diabetic patients in future.

Therefore, to conclude, uncontrolled hyperglycaemia and subsequently developed diabetes pose a significant medical and public health concerns worldwide. It is well-known that the incidence of diabetes are ever-increasing because of ageing societies and rates of obesity on a global scale. The research provided in this Editorial provides further evidence in support of these biological and clinical changes induced by poor glycaemic control, in addition to proposing improved prevention measures and interventions that may aid our efforts to mitigate the oral conditions and complications associated with such diabetic patient populations.
